# Hazard Characterization of Synthetic Cathinones Using Viability, Monoamine Reuptake, and Neuronal Activity Assays

**DOI:** 10.3389/fnins.2020.00009

**Published:** 2020-01-29

**Authors:** Anne Zwartsen, Michiel E. Olijhoek, Remco H. S. Westerink, Laura Hondebrink

**Affiliations:** ^1^Neurotoxicology Research Group, Toxicology Division, Institute for Risk Assessment Sciences (IRAS), Faculty of Veterinary Medicine, Utrecht University, Utrecht, Netherlands; ^2^Dutch Poisons Information Center (DPIC), University Medical Center Utrecht, Utrecht University, Utrecht, Netherlands

**Keywords:** bath salts, designer drugs, hazard characterization, synthetic cathinones, *in vitro* neurotoxicity assays

## Abstract

Synthetic cathinones are the second largest class of new psychoactive substances (NPS) on the drug market. Despite the large number of different cathinones and their abundant use, hazard characterization is mainly limited to their potential to inhibit monoamine transporters. To expand the current hazard characterization, we first investigated the acute effects of several synthetic cathinones [4-methylethcathinone (4-MEC), 3-methylmethcathinone (3-MMC), 4-MMC, methylone, pentedrone, α-pyrrolidinovalerophenone (α-PVP), and 3,4-methylenedioxypyrovalerone (MDPV)] on human dopamine, norepinephrine, and serotonin reuptake transporters (hDAT, hNET, and hSERT), which were stably transfected in human embryonic kidney (HEK) 293 cells. Next, we examined effects on spontaneous neuronal activity in rat primary cortical cultures grown on microelectrode arrays (MEAs) as an integrated endpoint for neurotoxicity. Changes in neuronal activity were assessed after acute (30 min) and prolonged (4.5 h) exposure. Moreover, we investigated whether neuronal activity recovered after washout of the exposure (24 h after the start of the 5 h exposure). Low micromolar concentrations of synthetic cathinones inhibited monoamine uptake via hDAT and hNET, while higher cathinone concentrations were needed to inhibit uptake via hSERT. Comparable high concentrations were needed to inhibit spontaneous neuronal activity during acute (30 min) and prolonged (4.5 h) exposure. Notably, while the inhibition of neuronal activity was reversible at low concentrations, only partial recovery was seen following high, but non-cytotoxic, concentrations of synthetic cathinones. Synthetic cathinones with either a pyrrolidine moiety or long alkyl-tail carbon chain more potently inhibit monoamine uptake via hDAT and neuronal activity. Monoamine uptake via hNET was most potently inhibited by synthetic cathinones with a pyrrolidine moiety. The combination of integrated measurements (MEA recordings of neuronal activity) with single target assays (monoamine reuptake transporter inhibition) indicates inhibition of hDAT and hNET as the primary mode of action of these synthetic cathinones. Changes in neuronal activity, indicative for additional mechanisms, were observed at higher concentrations.

## Introduction

Over the last decade, new psychoactive substances (NPS) have acquired a steady interest and place on the drug market. After the synthetic cannabinoids, synthetic cathinones are the most popular and abundant class of NPS on the drug market (United Nations Office on Drugs and Crime [UNODC], 2017). At the end of 2017, 148 synthetic cathinones were monitored by the United Nations Office on Drugs and Crime (United Nations Office on Drugs and Crime [UNODC], 2018).

Synthetic cathinones are derivatives from the phenylalkylamine cathinone, which is the naturally occurring stimulant in the khat plant (*Catha edulis*). Structurally, synthetic cathinones resemble amphetamine and 3,4-methylenedioxymethamphetamine (MDMA) with the addition of a ketone group (=0). The first synthetic cathinone derivative [i.e., methcathinone (MCAT)] was produced in the late 1920s (Glennon, 2014). Due to its abuse, MCAT was categorized as a Schedule 1 drug in 1971 by the United Nations’ Convention on Psychotropic Substances. Decades later, novel synthetic cathinones were introduced, starting with mephedrone (4-MMC) in 2006. In the following year, when mephedrone popularity peaked, novel cathinones methylone, 3,4-methylenedioxypyrovalerone (MDPV), α-pyrrolidinovalerophenone (α-PVP), pentedrone, 3-methylmethcathinone (3-MMC), and 4-methylethcathinone (4-MEC) were introduced to the drug market (Hondebrink et al., 2018; Majchrzak et al., 2018). The cathinone market peaked in 2014 when 30 novel synthetic cathinones were notified to the EU Early Warning System (European Monitoring Centre for Drugs and Drug Addiction [EMCDDA], 2018).

Desired and adverse effects of cathinones overlap with cocaine, amphetamine, and MDMA, and include mild to severe sympathomimetic toxicity and an altered mental status. Consequently, the use of cathinones has resulted in many emergency department visits, poisonings, and fatalities (Wiegand et al., 2012; Gunderson et al., 2013; Hondebrink et al., 2018). Anecdotal user reports suggest variation in effect profile between cathinones (Gunderson et al., 2013). Effects could also differ between drug batches, as the online advertised purity of >95% is not always reached (Gunderson et al., 2013).

Comparable to well-known illicit drugs, cathinones inhibit the reuptake of monoamines via the dopamine (DA), norepinephrine (NE), and/or serotonin (5-HT) reuptake transporters (DAT, NET, SERT) (Simmler et al., 2014; Hondebrink et al., 2018). The pharmacological and toxicological profile of cathinones is not fully elucidated, as hazard characterization is usually based solely on the effects of cathinones on these main targets. However, other targets like receptors and ion channels could also be affected by cathinones. Testing various (secondary) targets would increase knowledge on additional mechanisms of action, but would be time- and money consuming when using single target assays. In addition, toxicity may be over- or underestimated, as effects on other targets could mitigate or exacerbate effects. Therefore, applying an integrated method to measure the effects of cathinones on a diverse range of neuronal targets in a single assay could aid hazard characterization.

**FIGURE 1:**
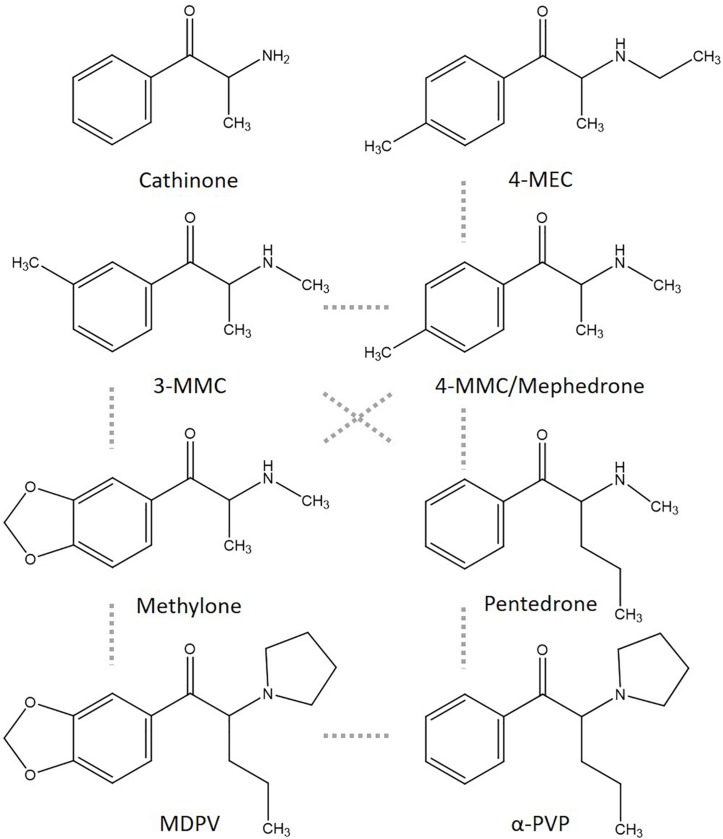
Chemical structures of cathinone and several cathinone derivatives. Structurally most comparable structures are linked by a dashed gray line.

In previous research, the applicability of neuronal cultures grown on microelectrode arrays (MEAs) as an efficient screening tool to determine the neurotoxicity of pharmaceuticals, toxins, illicit drugs, and NPS was shown (Dingemans et al., 2016; Vassallo et al., 2017; Strickland et al., 2018; Zwartsen et al., 2018, 2019). MEA measurements allow for determining effects of synthetic cathinones on spontaneous neuronal network activity, thereby including a range of neuronal targets in a single assay. Combining results from single target assays, like inhibition of monoamine reuptake transporters, with integrated methods, like inhibition of neuronal network activity, would strengthen hazard characterization of synthetic cathinones. In the present study, we therefore investigated the potencies of several synthetic cathinones to affect both the uptake via monoamine transporters and neuronal activity (for chemical structures see Figure 1).

## Materials and Methods

### Chemicals

3-Methylmethcathinone (2-(methylamino)-1-(3-methylphenyl) propan-1-one), 4-MMC (2-(methylamino)-1-(4-methylphenyl) propan-1-one), 4-MEC (2-(ethylamino)-1-(4-methylphenyl) propan-1-one), methylone (1-(1,3-benzodioxol-5-yl)-2-(methy lamino)propan-1-one), pentedrone (2-(methylamino)-1-phenyl pentan-1-one), and MDPV (1-(1,3-benzodioxol-5-yl)-2-pyrro lidin-1-ylpentan-1-one) hydrochloride salts (purity > 98.5%) were obtained from Lipomed (Weil am Rhein, Germany). Data on α-PVP were published previously (Zwartsen et al., 2017, 2019). 3-MMC and 4-MEC were also purchased from a commercial website for “research chemicals” and are described in this article as 3-MMC_internet_ and 4-MEC_internet_. Gas chromatography–mass spectrometry (GC–MS) analysis showed a purity of >99.5% of the online purchased drugs (see Supplementary Materials for methods and Supplementary Figure 1). All other chemicals were purchased from Life Technologies (Bleiswijk, Netherlands) unless otherwise stated. Stock solutions and dilutions were freshly prepared in HBSS (1×) at the day of usage.

### Effects on Monoamine Reuptake Transporters in Transfected HEK 293 Cells

#### Culture of Transfected HEK 293 Cells

Human embryonic kidney (HEK) 293 cells stably expressing human DAT, NET, or SERT (kindly provided by Dr. Hoener from F. Hoffmann-La Roche Ltd., Basel, Switzerland) were cultured as described in Zwartsen et al. (2017).

#### Monoamine Uptake Assay

Uptake activity of hNET, hDAT, and hSERT was measured using the Neurotransmitter Transporter Uptake Assay Kit from MDS Analytical Technologies (Sunnyvale, CA, United States) as described in detail previously (Zwartsen et al., 2017). The kit contained a mixture consisting of a fluorescent substrate, which resembles the biogenic amine neurotransmitters, and a masking dye that extinguishes extracellular fluorescence. Uptake of the fluorescent substrate, a measure of neurotransmitter transporter function, increases intracellular fluorescence, while extracellular fluorescence is blocked by the masking dye (Jorgensen et al., 2008). The mixture was dissolved in Hank’s Balanced Salt Solution (1×) (HBSS) with 20 mM HEPES, and kept up to 1 week at −20°C.

On day 0, HEK 293 cells were seeded (60,000 cells/well) in clear-bottom black-walled 96-well plates coated with poly-L-lysine (PLL, 50 mg/L). On day 1, cells were pre-incubated with the fluorescent substrate mixture (95 μL/well, comprising of the fluorescent substrate and the extracellular masking dye, dissolved in HBSS) for 12 min (*t* = −12 to *t* = 0). During incubation, intracellular fluorescence was measured every 3 min. After 12 min (at *t* = 0), 100 μL/well HBSS without (control) or with drug was added and uptake was continuously measured for 48 min to determine drug-induced inhibition of the monoamine transporters. Stock solutions and dilutions were freshly prepared in HBSS (1×) at the day of usage. Effects of 3-MMC, 4-MMC, 4-MEC, methylone, pentedrone, MDPV, 3-MMC_internet_, and 4-MEC_internet_ were measured at final concentrations of 0.03–300/1000 μM. Fluorescence was measured with a microplate reader (Tecan Infinite M200 microplate; Tecan Trading Männedorf, Switzerland) at 37°C at 430/515 nm excitation/emission wavelength. For more experimental detail see Zwartsen et al. (2017).

#### Uptake Analysis and Statistics

Data from the monoamine uptake assay were analyzed as described by Zwartsen et al. (2017), with minor modifications. Data for α-PVP were reanalyzed from Zwartsen et al. (2017). In short, the fluorescence of each well was background corrected and uptake was determined per well by calculating the change in fluorescence (ΔFU) at 12 min after drug exposure (*t* = 12) compared to the fluorescence at the start of exposure (*t* = 0), as a percentage of the fluorescence at the start of exposure (%Δ*FU* = ((FU*t* = 12−FU*t* = 0)/FU*t* = 0)∗100%; see Zwartsen et al., 2017). Plate-matched control values for each compound (e.g., methylone) above or below two times the standard deviation of the average normalized control value were considered outliers and were removed from analysis (4.8%). Thereafter, uptake in drug-exposed wells was expressed as a percentage of control wells on the same plate and all uptake values were scaled between 0 and 100%. Outliers (>mean ± 2×SD) in exposed groups were removed (2.7%) and uptake (% compared to control) was expressed as mean ± SEM of *n*_wells_ from *N*_plates._

#### Cell Viability of HEK 293 Cells

To exclude that effects of cathinones on transporter inhibition were due to a reduction in cell viability, cytotoxicity was investigated using a Neutral Red assay. At day 0, 100 μL of a cell suspension of 600,000 cells/mL was added to each well of a transparent 96-well plate (Greiner Bio-one, Solingen, Germany), coated with PLL. At day 1, medium was removed, and cells were exposed to 3-MMC, 4-MMC, 4-MEC, methylone, pentedrone, MDPV, 3-MMC_internet_, or 4-MEC_internet_ [final concentrations 10–1000 μM in HBSS (1×)]. After 48 min, the exposure medium was changed into DMEM culture medium before the plates were stored at 37°C, 5% CO_2_/95% air atmosphere until cell viability was tested 24 h after the start of exposure. At least 20 min before testing cell viability, several non-exposed wells were lysed to obtain background values.

Cell viability was determined as described in Zwartsen et al. (2019). In short, medium and lysis buffer were removed from all wells after which 100 μL NR solution (Invitrogen, Breda, Netherlands; 12 μM in phenol-red free NB-A medium w/o supplements) was added to the cells. Following 1 h incubation in the dark at 37°C, the solution was removed, and the cells were lysed using 100 μL NR lysis buffer. The plate, covered in aluminum foil, was placed on a plate shaker for ∼30 min before fluorescence was measured at 530/645 nm using the Tecan Infinite M200 microplate (Tecan Trading Männedorf, Switzerland). Cell viability was calculated according to Zwartsen et al. (2019), and expressed as mean ± SEM of *n*_wells_ from *N*_plates_. In total, 4.0% of the values were considered outliers (>mean ± 2×SD).

### Effects on Spontaneous Neuronal Network Activity Using Rat Cortical Cultures

#### Culture of Neuronal Networks Derived From Rat Cortices

Rat pups born of timed-pregnant Wistar rats (Envigo, Horst, Netherlands) were sacrificed on postnatal day 0–1 to prepare cortical cultures grown on MEA plates as described previously (Zwartsen et al., 2018, 2019). Briefly, a 50 μL drop of cell suspension was added to each well (1 × 10^5^ cells/well) of a 48-well MEA plate (Axion BioSystems Inc., Atlanta, GA, United States, M768-GL1-30Pt200) coated with 0.1% polyethyleneimine (PEI). After 2 h, 450 μL dissection medium was added to each well. The day after the isolation (day *in vitro* 1; DIV1), 450 μL/well dissection medium was replaced with 450 μL/well glutamate medium. At DIV4, 450 μL/well glutamate medium was replaced with 450 μL/well FBS medium (for medium supplements see Zwartsen et al., 2018, 2019). Cultures were kept in FBS medium at 37°C, 5% CO_2_/95% air atmosphere until use at DIV9-10.

#### MEA Recordings of Spontaneous Neuronal Network Activity

Microelectrode array recordings were performed as described in Zwartsen et al. (2019). In short, neuronal activity was measured using a Maestro 768-channel amplifier (Axion BioSystems Inc., Atlanta, GA, United States). Baseline spontaneous neuronal activity was recorded for 30 min at 37°C, after which wells were exposed to selected synthetic cathinones under sterile conditions. Next, neuronal activity was determined during a 30 min “acute exposure” recording. As the half-life of most illicit drugs and NPS *in vivo* ranges from 0.5 to 5 h in plasma [see Zwartsen et al. (2019) for references], the plate was subsequently incubated for an additional 4 h of exposure at 37°C, after which activity was measured during a 30 min “prolonged exposure” recording. Next, exposure medium was replaced with fresh FBS medium and the plate was incubated for 19 h at 37°C, until the 30 min “washout” recording, i.e., 24 h after the start of the exposure.

Effects of 3-MMC, 4-MMC, 4-MEC, pentedrone, 3-MMC_internet_, and 4-MEC_internet_ were tested at 1–1000 μM (final concentration). At the day of exposure, stocks and dilutions were freshly made in NB-A FBS medium (for medium supplements see Zwartsen et al., 2019). Data on methylone, α-PVP, and MDPV exposure have been published previously in Zwartsen et al. (2019). For each experimental condition, primary cultures from two to three different isolations were used and tested in four to five plates (*N*_plates_). The number of wells (*n*_wells_) represents the number of replicates per condition.

#### MEA Analysis

Microelectrode array data were analyzed as described in Zwartsen et al. (2019). In short, parameters of interest after acute exposure were expressed as a percentage of the parameters prior to exposure to obtain a treatment ratio for each well (paired comparison; parameter_exposure_/parameter_baseline_ as % of control wells). The parameters after prolonged exposure and washout were also expressed as a percentage of the baseline parameters. Next, treatment ratios were grouped per parameter, condition, drug [e.g., mean spike rate (MSR) 10 μM 3-MMC] and exposure scenario (acute, prolonged, or washout).

Outliers (>mean ± 2×SD) for MSR (5.0%) were used to exclude wells on all parameters. Outliers for mean burst rate (MBR; 2.6%) and mean network burst rate (MNBR; 1.4%) were used to exclude wells on specific parameters (burst, network burst, and synchronicity parameters, or network burst and synchronicity parameters, respectively). Finally, the acute, prolonged, and washout control treatment ratios were set to 100% and treatment ratios of exposed wells were normalized to the average treatment ratio of medium control wells of the corresponding parameter and exposure scenario. Treatment ratios of exposed wells were averaged per parameter (e.g., MSR), condition (e.g., 100 μM), drug (e.g., methylone), and exposure scenario (e.g., acute exposure) and used for further statistical analyses [see Zwartsen et al. (2019) for more details on criteria, parameters, and exposure scenarios]. Neuronal activity (as % of control) is expressed as mean ± SEM of *n*_wells_ from *N*_plates_. As the spike, burst, and network burst rates were among the most sensitive parameters, these parameters are presented in the manuscript. Additional parameters are presented in Supplementary Figure 2.

#### Cell Viability of Neuronal Cultures

To exclude that effects of cathinones on neuronal activity were due to cytotoxicity, cell viability was investigated as described in the section “Cell Viability of HEK 293 Cells,” with minor modifications. Briefly, 100 μL of a cell suspension of rat cortical cells (3.0 × 10^4^ cells/well) was added to each well of a transparent 96-well plate (Greiner Bio-one, Solingen, Germany). Medium was changed at DIV1 and DIV4 as described for the 48-wells MEA plates, only at smaller volumes (100 μL/well). In addition, the glutamate to FBS medium change on DIV4 was done using phenol-red free FBS medium (medium supplements described in Zwartsen et al., 2019). At DIV9-10, cells (three to six plates from two to three different primary cultures) were exposed for 4.5 h to 3-MMC, 4-MMC, 4-MEC, pentedrone, 3-MMC_internet_, and 4-MEC_internet_ (final concentrations 1–1000 μM in phenol-red free NB-A FBS medium). Thereafter, the exposure medium was replaced with phenol-red free FBS medium before the plates were stored at 37°C, 5% CO_2_/95% air atmosphere until the cell viability was tested 19.5 h later, 24 h after the start of exposure. Neutral Red cell viability assay was performed and analyzed as described in Zwartsen et al. (2019). Following the exclusion of outliers (>mean ± 2×SD; 5.4% in the normalized control values and 4.8% in the experimental conditions), cell viability was expressed as mean ± SEM of *n*_wells_ from *N*_plates_.

### Statistical Analysis

Concentration–response curves were made for transporter, MEA, and cell viability assays. To calculate IC_50_ values, a four-parameter logistic curve with a variable slope was used (*Y* = Bottom + (Top−Bottom)/(1 + 10∧((LogIC_50_ − X) ^∗^ HillSlo pe))) (GraphPad Prism, version 7.04). When applicable, significance between concentrations and controls (for MEA) and different IC_50_ values [between different transporters (DAT/NET/SERT), MEA parameters (MSR/MBR/MNBR), exposure settings (acute/prolonged/recovery), and drug grade (pharmaceutical/internet)] were determined using unpaired *t*-tests (two values) or one-way ANOVA’s followed by a *post hoc* Dunnet’s test (>2 values). All statistical tests were performed using GraphPad Prism. For recovery of neuronal activity, IC_50_ values and 95% confidence intervals could not always be determined and are reported as higher than the highest concentration tested (e.g., >1000 μM). In these cases, statistical analysis comparing activity following recovery to activity during acute or prolonged exposure could not be performed. Effects on monoamine reuptake, neuronal activity, and cell viability were considered relevant when the effect was statistically significant (*p* < 0.05) and above the biological variation of the control values (≥15, ≥30, and ≥10%, respectively).

### Estimated Brain Concentrations

To correlate reported effect concentrations to expected concentrations in the human brain during recreational use a brain partitioning factor (BPF; serum/brain ratio) was applied, as actual human brain concentrations following recreational drug use are unknown. Expected concentrations in the human brain were estimated by multiplying recreational human blood/serum/plasma concentrations with the BPF. Human recreational blood, serum, or plasma levels were obtained from literature (driving under the influence or accidental non-fatal intoxications). The BPF was determined for each drug by dividing the brain concentration by the blood/serum/plasma concentration found in human post mortem reports, or animal studies (rat/mice) when human data were insufficient.

## Results

### Effects on Monoamine Reuptake

**FIGURE 2:**
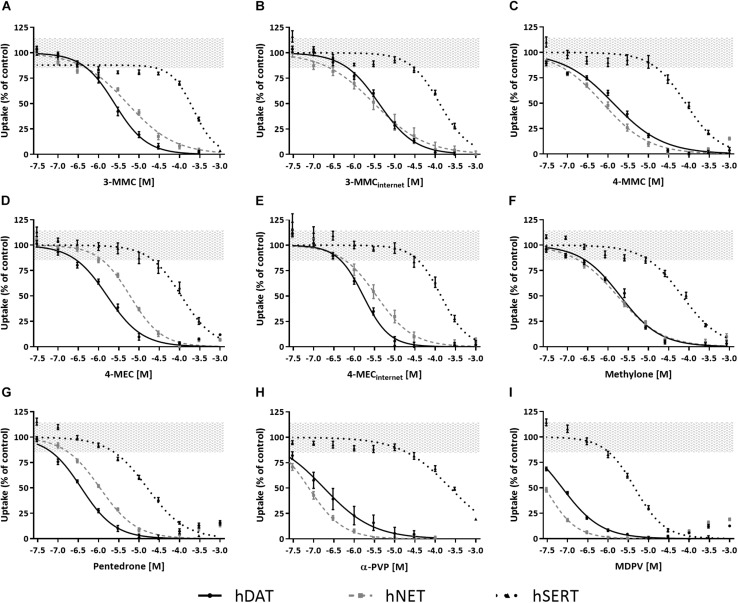
Concentration–response curves of synthetic cathinones for the inhibition of uptake of fluorescent substrate via monoamine transporters. The inhibition of uptake via hDAT (solid line), hNET (dashed gray line), and hSERT (dotted line) was tested for 3-MMC **(A)**, 3-MMC_internet_ **(B)**, 4-MMC **(C)**, 4-MEC **(D)**, 4-MEC_internet_ **(E)**, methylone **(F)**, pentedrone **(G)**, α-PVP **(H)**, and MDPV **(I)**. Uptake is shown as mean ± SEM percentage compared to control (*n*_wells_ = 13–41, *N*_plates_ = 4–5). Effects ≤ 15% (i.e., the variation of medium control) are considered not to be of (toxicological) relevance, which is depicted by the gray area. The corresponding IC_50_ values can be found in Table 1. α-PVP was reanalyzed from previously published data (Zwartsen et al., 2017).

All cathinones inhibited monoamine uptake via hDAT, hNET, and hSERT (Figure 2; data for α-PVP re-analyzed from Zwartsen et al., 2017). Uptake via hSERT was less potently inhibited compared to uptake via hDAT and hNET. 3-MMC, 4-MEC, and pentedrone inhibited hDAT at lower concentrations compared to hNET, while 4-MMC, α-PVP, and MDPV more potently inhibited hNET (*p* < 0.05; Table 1). Methylone inhibited hDAT and hNET with comparable potency. MDPV was the most potent hDAT and hNET inhibitor with IC_50_ values of 0.07 and 0.03 μM, respectively (Table 1). For hDAT inhibition the rank order was MDPV < α-PVP/pentedrone < 4-MMC/4-MEC < methylone/3-MMC. hNET was inhibited with the following rank order: MDPV < α-PVP < 4-MMC < pentedrone/methylone < 3-MMC/4-MEC. Uptake via hSERT was inhibited by MDPV and pentedrone with IC_50_ values of 4.5 and 16 μM, respectively, while all other cathinones inhibited hSERT with IC_50_ values ≥100 μM (Table 1).

**TABLE 1:** IC_50_ values for the inhibition of neuronal activity (MSR, MBR, and MNBR) and monoamine reuptake transport (hDAT, hNET, and hSERT) of cathinones.

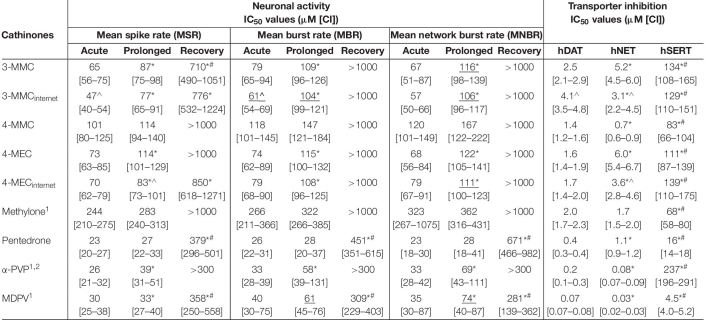

Neuronal activity: Following recovery, several IC_50_ values and 95% confidence intervals could not be determined and were depicted as higher than highest concentration tested (e.g., >1000 μM). * depicts IC_50_ values of prolonged exposure or following washout that differ significantly from acute exposure (*p* < 0.05). # depicts IC_50_ values following washout that differ significantly from prolonged exposure (*p* < 0.05). IC_50_ values for MBR and MNBR significantly different from MSR at the same exposure scenario (acute, prolonged, or recovery) are underlined (*p* < 0.05; on “>” values statistics could not be performed). IC_50_ values for MNBR and MBR at the same exposure scenario (acute, prolonged, or recovery) did not significantly differ (*p* < 0.05). Transporter inhibition: * depicts hNET or hSERT IC_50_ values significantly different from hDAT IC_50_ values (*p* < 0.05). ^#^ depicts hSERT values significantly different from hNET IC_50_ values (*p* < 0.05). Internet vs. pharmaceutical grade cathinones: ^∧^ depicts IC_50_ values from cathinones bought via the internet different from pharmaceutical grade IC_50_ values (*p* < 0.05). ^1^Neuronal activity data from Zwartsen et al. (2019). ^2^Transporter inhibition data reanalyzed from Zwartsen et al. (2017).

While 4-MEC_internet_ inhibited hDAT with comparable potency compared to pharmaceutical grade 4-MEC, less than twofold differences in potencies between the pharmaceutical grade and internet-bought drugs for 4-MEC at hNET and 3-MMC at hDAT and hNET were seen (*p* < 0.05; Figures 2B,E and Table 1). Inhibition of uptake via hSERT did not significantly differ between the pharmaceutical grade cathinones and the cathinones bought via the internet.

### Effects on Neuronal Activity

**FIGURE 3:**
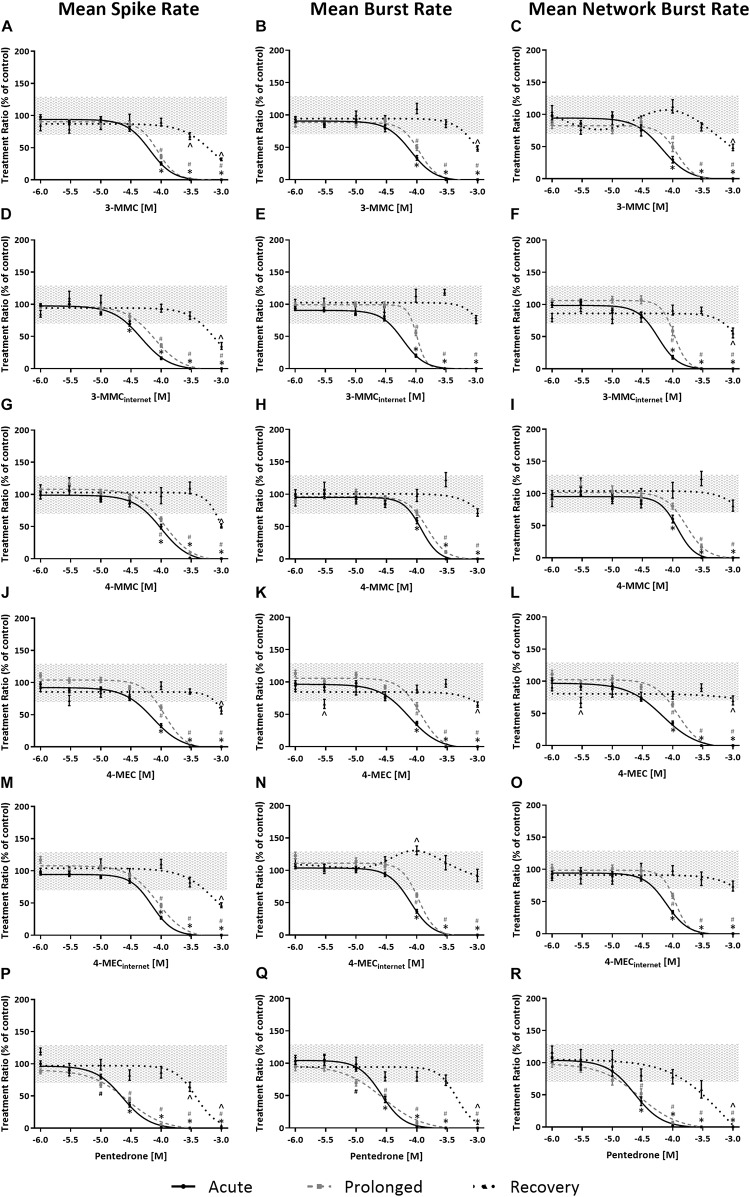
Concentration–response curves of synthetic cathinones for neuronal activity. The mean spike rate (MSR), mean burst rate (MBR), and mean network burst rate (MNBR) after acute exposure (solid line, 30 min), prolonged exposure (dashed gray line, 4.5 h) and 19 h washout (dotted line, 24 h from the start of exposure) are shown for 3-MMC **(A–C)**, 3-MMC_*internet*_ **(D–F)**, 4-MMC **(G–I)**, 4-MEC **(J–L)**, 4-MEC_*internet*_ **(M–O)**, and pentedrone **(P–R)** (*n*_*wells*_ = 8–31, *N*_*plates*_ = 4–5). Neuronal activity is depicted as the mean treatment ratio ± SEM (parameter_*exposure*_/parameter_*baseline*_ as % of control wells). Effects ≤ 30% (i.e., the variation of medium control) are considered not to be of (toxicological) relevance, which is depicted by the gray area. Relevant effects that are statistically different from control (*p* < 0.05) are indicated with ^∗^ for acute exposure, ^#^ for prolonged exposure and ∧ for washout.

Effects of methylone, α-PVP, and MDPV were published previously in Zwartsen et al. (2019) (Supplementary Figure 2). Here, we extend on these results by measuring the effects of 3-MMC, 4-MMC, 4-MEC, and pentedrone on neuronal activity. All investigated synthetic cathinones inhibited the MSR, MBR, and MNBR after acute exposure (Figure 3; for other parameters and heatmaps see Supplementary Figure 2). Based on IC_50_ values, no significant difference in sensitivity was seen between MSR, MBR, and MNBR (Table 1). Of the four synthetic cathinones, pentedrone most potently inhibited neuronal activity during acute exposure (MSR IC_50_ value of 23 μM), followed by 3-MMC, 4-MEC, and 4-MMC (MSR IC_50_ values of 65, 73, and 101 μM, respectively, Table 1). For most synthetic cathinones, neuronal activity was slightly less inhibited following prolonged exposure compared to acute exposure. Following washout, neuronal networks partially or fully recovered from exposure. However, neuronal networks exposed to the highest concentration of pentedrone remained fully inhibited.

During acute exposure, 3-MMC_internet_ inhibited the MSR and MBR, yet not the MNBR, significantly more potently compared to pharmaceutical grade 3-MMC (Figures 3D–F and Table 1). During prolonged exposure, no significant differences were seen between the pharmaceutical grade 3-MMC and the 3-MMC_internet_. The potency of 4-MEC_internet_ did not significantly differ from the potency of pharmaceutical grade 4-MEC to inhibit MSR, MBR, or MNBR at any exposure scenario (except for the IC_50_ for the MSR during prolonged exposure) (Figures 3M–O).

### Cell Viability

In general, no cytotoxicity was seen in HEK 293 cells stably transfected with hDAT, hNET, and hSERT or rat primary cortical cultures after exposure to ≤1000 μM pharmaceutical grade cathinones or cathinones bought via the internet (Supplementary Figures 3, 4).

## Discussion

Toxicological profiles used for hazard characterization of synthetic cathinones are usually based on single target assays (i.e., specific receptors, ion channels, or transporters). As such, the current study aimed at strengthening the hazard characterization of synthetic cathinones (Figure 1) by combining effects obtained with a single target assay (monoamine reuptake; Figure 2) and those obtained with an integrated assay (neuronal activity; Figure 3). To enlarge the data set, monoamine transporter data for α-PVP and MEA data for methylone, α-PVP, and MDPV from previous publications have been included (Table 1).

Our results confirm previous reports on the potent inhibitory effects of cathinones on monoamine transporters hDAT and hNET, and the less potent inhibition of hSERT [Table 1; for review see Hondebrink et al. (2018); also see Baumann et al., 2013; Eshleman et al., 2013; Simmler et al., 2013; Kolanos et al., 2015; Saha et al., 2015; McLaughlin et al., 2017]. The magnitude of the hSERT IC_50_ values for the cathinones without pyrrolidine structure or lengthened alkyl chain (3-MMC, 4-MMC, 4-MEC, and methylone) showed some deviations compared to literature. These differences are probably due to the use of different assays. Instead of using the traditional assays that rely on radioactively labeled monoamines, we used a fluorescent-based assay that relies on a fluorescent substrate, which resembles the biogenic amine neurotransmitters. While this is a relatively new assay, it has advantages over the traditional assays, primarily related to allowing for kinetic measurements at more physiological settings. For example, we measured effects at a physiological temperature (37°C), whereas experiments using radioactively labeled monoamines are mostly performed at room temperature. This can affect the results since the uptake of and binding to hSERT is temperature dependent (Elfving et al., 2001; Saldana and Barker, 2004; Oz et al., 2010; Tsuruda et al., 2010). For example, decreasing the temperature from 37°C to room temperature increases the potency of MDMA to inhibit hSERT threefold (Zwartsen et al., 2017). Possibly, a lower temperature also increases the potency of synthetic cathinones, including 3-MMC, 4-MMC, 4-MEC, and methylone for which we obtained higher IC_50_ values on hSERT compared to literature.

Furthermore, we determined monoamine uptake using a monolayer of interconnected cells, while in radioactive assays cells in suspension are often used. The process of obtaining cells in suspension can cause changes in cell morphology and damage to membrane proteins, resulting in cellular dysfunction and stress responses (Huang et al., 2010). This may increase the sensitivity of cells to the effect of toxicants, as was reported by Simmler and Liechti (2017), who stated that drugs were less potent releasers when attached cells were used compared to cells in suspension.

Notably, only one study investigated effects of recreational drugs on hSERT using radioactively labeled monoamines at 37°C while using attached cells. In line with our data, this study also reported lower potencies for amphetamine, 4-fluoroamphetamine, and MDMA to inhibit hSERT (Rosenauer et al., 2013). Similar to our data, for other compounds no differences in potencies were observed between both methods when experimental conditions like temperature and cell attachment were comparable (Jorgensen et al., 2008; Tsuruda et al., 2010).

Moreover, the fluorescent-based assay can detect potent hSERT inhibition, since we observed an IC_50_ value of 0.1 μM for fluoxetine. The fact that the fluorescent-based assay effectively detects SERT inhibition by this selective serotonin reuptake inhibitor (SSRI, Zwartsen et al., 2017) highlights the usability of the fluorescent-based assay. Which assay (radioactive of fluorescent-based) approximates the effects actually occurring in humans best remains to be determined.

Although the dataset is too narrow to perform an extensive structure–activity analysis, the presence of a pyrrolidine moiety, as present in MDPV and α-PVP, increases the potency to inhibit uptake via hNET and hDAT (*p* < 0.05), in line with other studies (Kolanos et al., 2013; Marusich et al., 2014). In addition, the lengthened alkyl-tail carbon chain (α-carbon side chain) also appears to increase the potency to inhibit uptake via hDAT, as seen by the IC_50_ values of pentedrone, α-PVP, and MDPV compared to 4-MMC, 4-MEC, and methylone. This is in line with research published by others (Marusich et al., 2014; Eshleman et al., 2017). Interestingly, Eshleman et al. (2017) reported a 100-fold increased affinity for hDAT when the alkyl-tail carbon chain changed from one to five carbons. This is possibly due to the increase of lipophilicity and molecular size of the lengthened drugs (Kolanos et al., 2015).

As this study is the first to describe the effects of cathinones on spontaneous neuronal activity, comparisons to literature could only be based on non-cathinone substances. Cathinones without a lengthened chain or methylenedioxy group (3-MMC, 4-MMC, and 4-MEC) showed comparable potency to inhibit neuronal activity following acute and prolonged exposure to and after washout from amphetamine-type stimulants MDMA, PMMA, and methamphetamine (Zwartsen et al., 2019). While the chemical structure of methylone and MDMA only differs in the addition of a keton group for methylone, this reduces the potency to inhibit neuronal activity threefold (Zwartsen et al., 2019). Cathinones with a lengthened alkyl chain (as present in pentedrone, α-PVP, and MDPV) show comparable potencies to inhibit neuronal activity to 2C-B, a hallucinogenic phenethylamine of the 2C family (Zwartsen et al., 2019).

In line with the monoamine reuptake transporters pentedrone, α-PVP, and MDPV had a two to fourfold increased potency to inhibit the neuronal activity (MSR, MBR, and MNBR) following both acute and prolonged exposure, compared to cathinones lacking the lengthened alkyl chain. In addition, increased neurotoxicity was observed for these pentedrone, α-PVP, and MDPV, as illustrated by the lack of (full) recovery of neuronal activity (Table 1; Zwartsen et al., 2019). As cortical cultures have a low expression of monoamine transporters (Dahlin et al., 2007), this lack of recovery of neuronal activity could suggest the involvement of additional neurotoxic mechanisms of cathinones.

We also investigated the effects of two cathinones bought via the internet (3-MMC_internet_ and 4-MEC_internet_) on neuronal activity and monoamine reuptake transporters. Effects on hSERT and neuronal activity did not differ between the pharmaceutical grade and internet-bought drugs. Between 4-MEC and 4-MEC_internet_, small differences were observed on hDAT and between 3-MMC and 3-MMC_internet_ small differences were observed for both hDAT and hNET. These differences all concerned a less than twofold change in potency likely due to biological or technical variation. Contaminations of the cathinones bought online is unlikely as GC–MS analysis showed 0≤0.5% contamination by structurally related compounds (>99.5% purity; see Supplementary Figure 1).

To identify the relevance of effects and potentially harmful drugs, effect concentrations (IC_50_ values) were related to the estimated human brain concentration during recreational use [(brain), Table 2]. By comparing these concentrations, the most relevant neurotoxic mechanisms of cathinones can be identified. All synthetic cathinones inhibited uptake via hDAT and hNET within the estimated human brain concentrations, while methylone and MDPV also inhibited hSERT at concentrations relevant for human exposure (Table 2). Only 4-MEC and MDPV inhibited neuronal activity during acute exposure at concentrations within (MDPV) or close to (4-MEC) the estimated brain concentration. Although the effect concentrations of MDPV and 4-MEC for neuronal activity are far above those of the transporters, this suggests that additional neurotoxic mechanisms could be at play during recreational drug use.

**TABLE 2:** Estimated human brain concentrations of cathinones compared to the monoamine reuptake transporter and neuronal activity inhibition potencies.

**Cathinones**	**Human (serum/blood/plasma) (μM)**	**Brain partitioning factor (BPF)**	**Estimated human (brain) (μM)**	**IC_50_ value at or close to estimated human (brain)**
3-MMC	0.005–9.0^A,b,^**^c^**	x	0.005–9.0	hDAT > hNET
4-MMC	0.05–6.0**^a^**^,d,E,f^	0.7–6.2^1,^**^–2,^^3^**	0.04–37	hNET > hDAT
4-MEC	0.1–4.7**^a^**^,d^	1.5–6.2^4^	0.2–29	hDAT > hNET >> MSR_a_, MBR_a_, MNBR_a_
Methylone	0.01–18^a,d,g,h^	1.4–4.5^5,^**^–6,^^7^**	0.01–81	hNET, hDAT >> hSERT
Pentedrone	0.04–1.9**^a^**^,d,I^	1.6^8^	0.06–3.0	hDAT > hNET
α-PVP	0.01–2.8^b,j,K^	0.1–1.8^4,8,9,10^	0.001–5.0	hNET > hDAT
MDPV	0.02–6.9^d,L,M,n^	0.8–4.4^4,11,12^	0.02–30	hNET > hDAT >> hSERT >> MSR_a_, MSR_p_, MBR_a_, MNBR_a_

*Estimated brain concentrations were calculated using human blood, serum (caps), or plasma (bold) concentrations and brain partition factors (BPF) found in literature. All human concentrations were obtained from reports on recreational use (voluntary intake, driving under the influence or accidental non-fatal intoxications). BPFs were based on blood/serum (underlined)/plasma (strikethrough) and brain concentrations of human or rat (bold) data. Estimated brain concentrations for 3-MMC were based on the observation that most BPFs are >1. Small to large differences in effect concentrations for different endpoints are depicted as > or >>, respectively. hDAT, human dopamine reuptake transporter; hNET, human norepinephrine reuptake transporter; hSERT, human serotonin reuptake transporter; MSR_*a*_, mean spike rate during acute exposure; MBR_*a*_, mean burst rate during acute exposure; MBNR_*a*_, mean network burst rate during acute exposure; MSR_*p*_, mean spike rate during prolonged exposure. References for serum concentrations: ^*a*^Turcant et al. (2017), ^*b*^Adamowicz et al. (2016b), ^*c*^Backberg et al. (2015), ^*d*^Elliott and Evans (2014), ^*e*^Wood et al. (2010), ^*f*^Cosbey et al. (2013), ^*g*^deRoux and Dunn (2017), ^*h*^Cawrse et al. (2012), ^*i*^World Health Organisation [WHO] (2016), ^*j*^Adamowicz et al. (2016a), ^*k*^Beck et al. (2016), ^*l*^Kriikku et al. (2011), ^*m*^Thornton et al. (2012), ^*n*^Marinetti and Antonides (2013). References for BPF calculation: ^1^Gerace et al. (2014), ^2^Hadlock et al. (2011), ^3^Martinez-Clemente et al. (2013), ^4^Lehmann et al. (2018), ^5^deRoux and Dunn (2017), ^6^Lõpez-Arnau et al. (2013), ^7^Štefková et al. (2017), ^8^Sykutera et al. (2015), ^9^Hasegawa et al. (2014), ^10^Potocka-Banas et al. (2017), ^11^Wyman et al. (2013), ^12^Marinetti and Antonides (2013).*

As neuronal activity is inhibited at concentrations several magnitudes above inhibition of uptake via hDAT and hNET, the monoamine transporters are a more sensitive endpoint for the cathinones tested. While this is true for cathinones, we have previously shown that neuronal activity is a more sensitive endpoint for other drugs like methoxetamine (MXE) and hallucinogenic phenethylamines 2C-B and multiple NBOMes (Hondebrink et al., 2017; Zwartsen et al., 2017, 2018). As neuronal activity reflects many neuropharmacological targets of interest, in contrast to single targets like monoamine transporters, these measurements can be very valuable in screening for neuropharmacological effects of NPS, especially when the mechanism of action is *a priori* unknown.

To conclude, by combining results from different methods to investigate the neurotoxicity of a (group of) drug(s), the most sensitive drug target can be determined. Inhibition of monoamine reuptake transporters is the most sensitive target for this set of synthetic cathinones, although the inhibition of neuronal activity at concentrations relevant for recreational exposure indicated the possible relevance of additional targets for some cathinones. Therefore, hazard characterization of emerging NPS can be optimized by applying several *in vitro* screening methods directed to different specific or integrated targets.

## Data Availability Statement

The datasets generated for this study are available on request to the corresponding author.

## Ethics Statement

Animal experiments were performed in agreement with Dutch law, the European Community directives regulating animal research (2010/63/EU) and reviewed and approved by the Ethical Committee for Animal Experiments of Utrecht University. All efforts were made to minimize the number of animals used and their suffering.

## Author Contributions

AZ, RW, and LH designed the study. AZ and MO performed the experiments. AZ performed the statistical analysis. AZ wrote the first draft of the manuscript. All authors contributed to the manuscript revision, and read and approved the submitted version of the manuscript.

## Conflict of Interest

The authors declare that the research was conducted in the absence of any commercial or financial relationships that could be construed as a potential conflict of interest.
