# Supplementary material for: Hazard Characterization of Synthetic Cathinones Using Viability, Monoamine Reuptake, and Neuronal Activity Assays
Source: Front Neurosci. 2020 Jan 29;14:9. doi: 10.3389/fnins.2020.00009 (PMC7000521; doi:10.3389/fnins.2020.00009)
Supplement: Supplementary file 1 [file Table_1.DOCX]

**Supplemental material**

**Purity of new psychoactive substances (NPS) bought on the internet**

**To compare the effects of pharmaceutical grade cathinones and cathinones as sold on the drug market, we bought 1 gram 4-MEC and 1 gram 3-MMC online via a commercial website for ‘research chemicals’.**

**The purity of these NPS was determined using gas chromatography-mass spectrometry (Agilent 5977A MSD). Drugs were solved in methanol and 1 µl was injected in split mode (split ratio of 100) at 225 °C. Column used was a SGE HT8 50m x 0.22 mm with 0.25 um film thickness. Helium was used as carrier gas with a flow of 1 ml/min. GC temperature program was as follows: 150 °C-1 minute- 10 °C/min.- 300 °C -5 minutes. MS was used in the scan mode 40-250 AMU.** Purity was >99.5% for 4-MEC_internet_ and ~99.5% for 3-MMC_internet_ (**Supplementary Fig. 1**). Both drugs were assumed to be conjugated to hydrochloride salts.


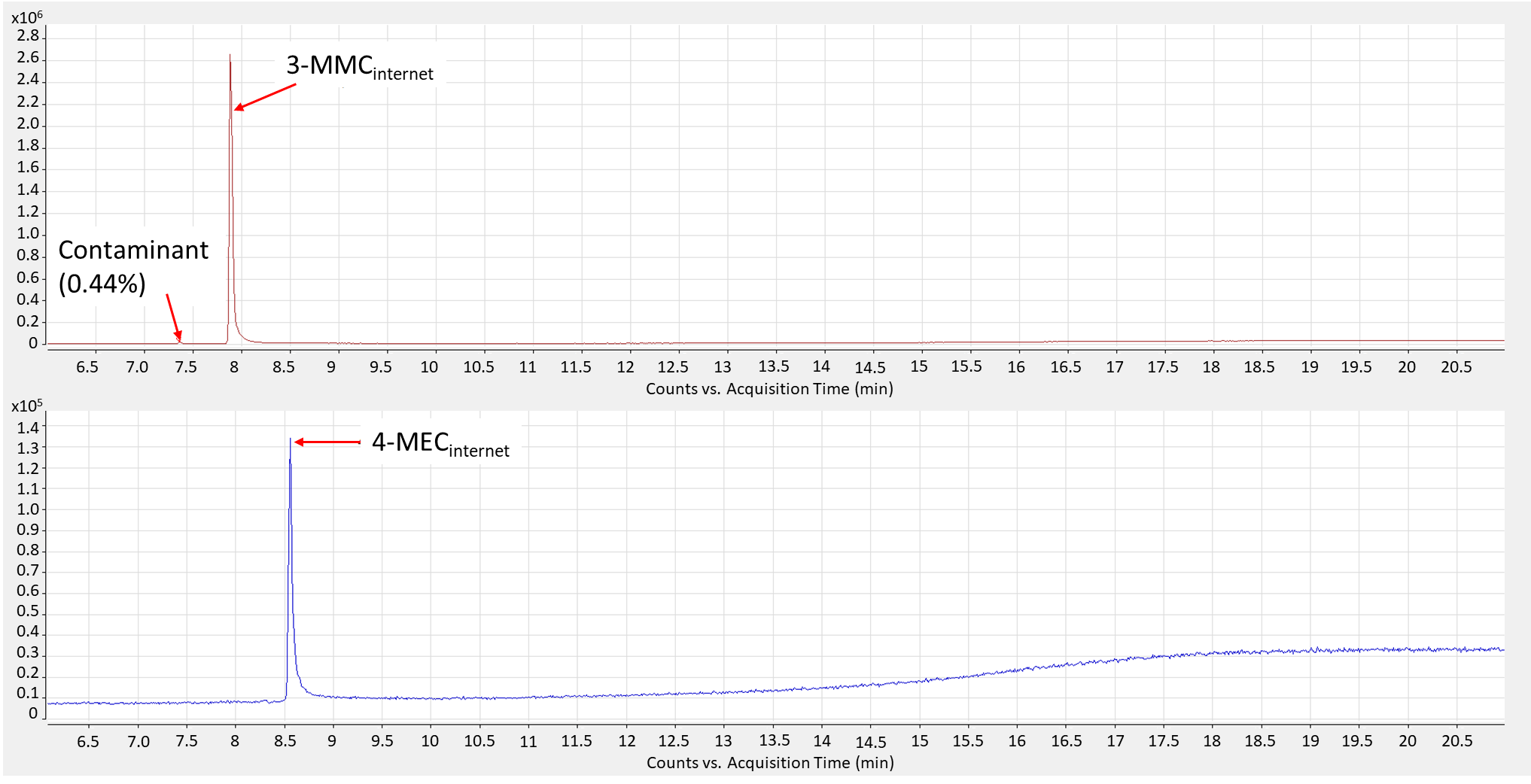


**Supplementary Fig. 1.** GC-MS trace of 3-MMC (top) and 4-MEC (bottom) bought via the internet. Purity was determined with GC-MS to be ~99.5% for 3-MMC_internet_ and >99.5% for 4-MEC_internet_.

**Supplementary Fig. 2.** Heat map of the effects of cathinones 3-MMC, 4-MMC, 4-MEC, methylone, pentedrone, α-PVP and MDPV on selected parameters of neuronal activity**.** Colors depict the mean treatment ratio (parameter_exposure_/parameter_baseline_ as % of control wells) for the various metric parameters (for overview and description see supplemental table 1 in Zwartsen et al., 2019. Effects ≤ 30% (*i.e.* the variation of medium control) are considered not to be of (toxicological) relevance and are depicted in white. Parameters for which no value could be calculated due to complete inhibition of spike, burst or network bursts, are depicted with an X. Data from methylone, α-PVP and MDPV were reproduced from Zwartsen e*t al.* 2019.

**Zwartsen, A., Hondebrink, L., Westerink, R.H.S (2019). Changes in neuronal activity in rat primary cortical cultures induced by illicit drugs and new psychoactive substances (NPS) following prolonged exposure and washout to mimic human exposure scenarios. NeuroToxicology 74, 28-39.**


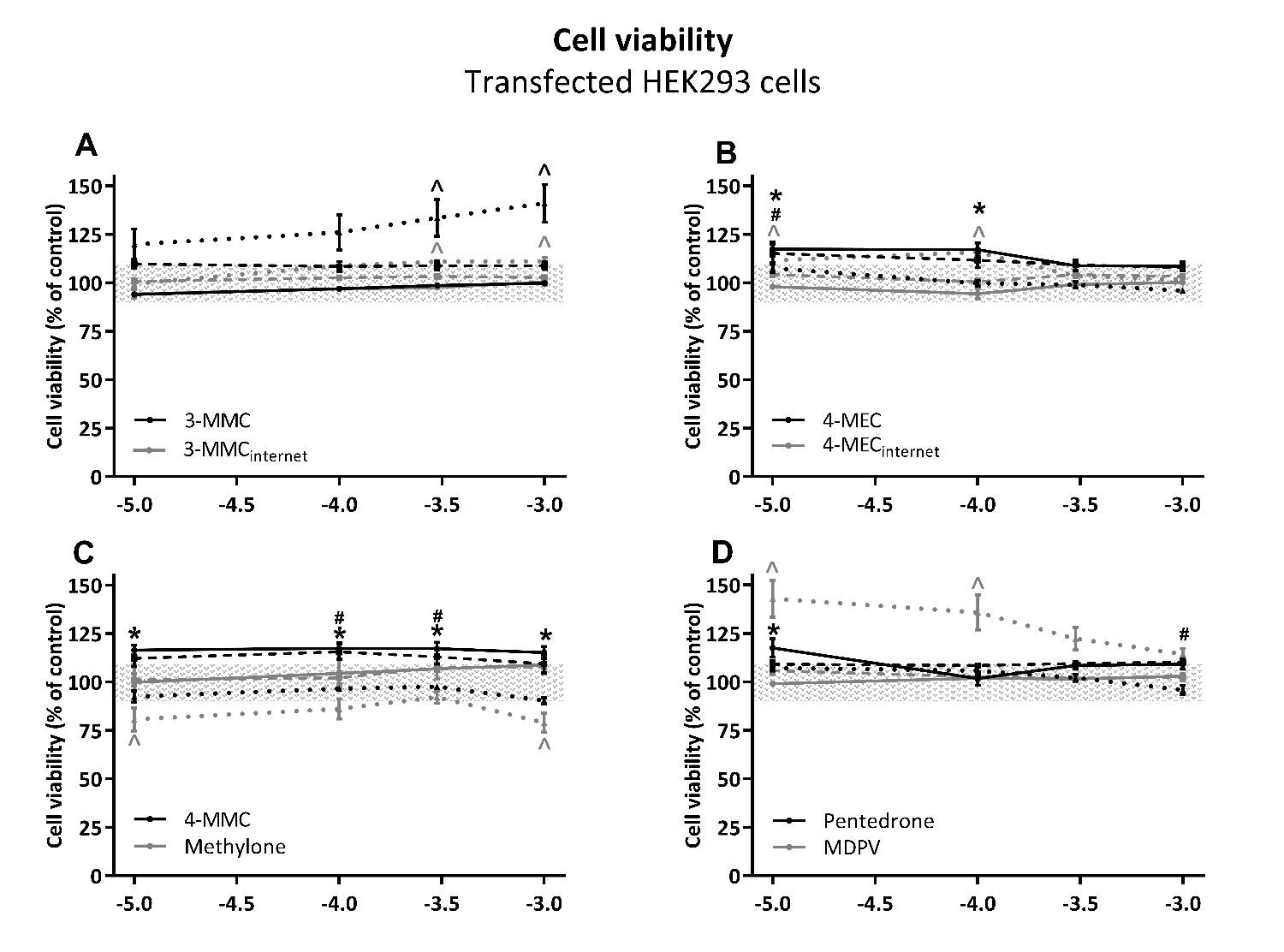


**Supplementary Fig. 3.** Cell viability of stably transfected HEK 293 cells after exposure to cathinones 3-MMC, 4-MEC, 4-MMC, methylone, pentedrone and MDPV. HEK 293 cells transfected with hDAT (solid line), hNET (dashed line) and hSERT (dotted line) were exposed to 10-1000 µM pharmaceutical grade and internet-bought cathinones (n_wells_=16-18, N_plates_=3) for 48 min, after which the exposure was removed, and fresh medium was added. 24 h after the start of exposure, cell viability was measured using the Neutral Red assay (See Methods 2.3.4 *Cell viability of HEK 293 cells*). Effects ≤ 10% (*i.e.* the variation of medium control) are considered not to be of (toxicological) relevance, which is depicted by the grey area. Relevant effects that are statistically different from control (*p*<0.05) are indicated with * for hDAT exposure, ^#^ for hNET exposure and ^ for hSERT (in black or grey).


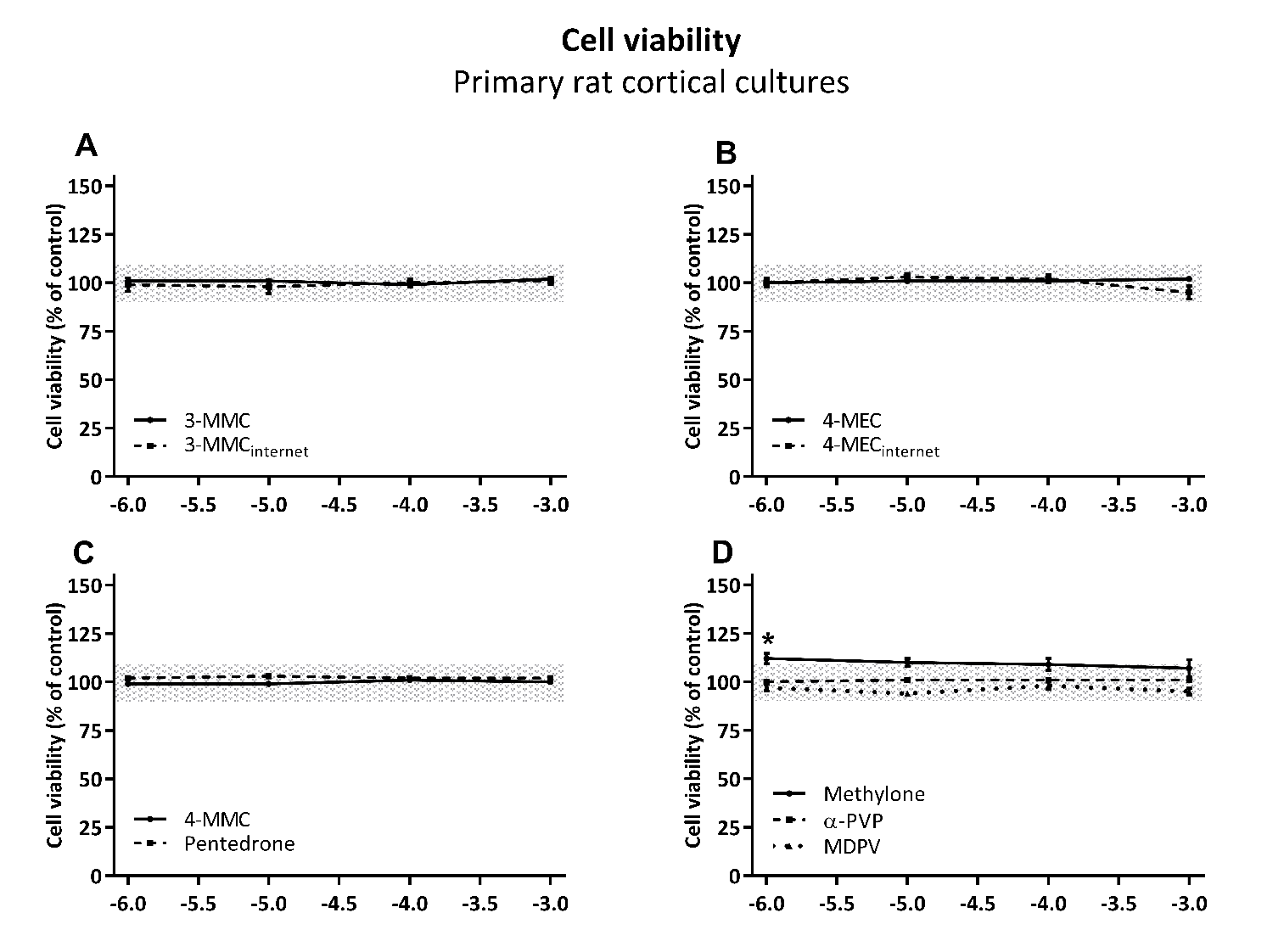


**Supplementary Fig. 4.** Cell viability of rat primary cortical cultures after exposure to cathinones 3-MMC, 4-MEC, 4-MMC, pentedrone, methylone, α-PVP and MDPV. Neuronal cultures were exposed to 1-1000 µM pharmaceutical grade and internet-bought cathinones (n_wells_=22-35, N_plates_=3-6) for 4.5 h, after which the exposure was removed, and fresh medium was added. 24 h after the start of exposure, cell viability was measured using the Neutral Red assay (See Methods 2.3.4 *Cell viability of neuronal cultures*). Effects ≤ 10% (*i.e.* the variation of medium control) are considered not to be of (toxicological) relevance, which is depicted by the grey area. Relevant effects that are statistically different from control (*p*<0.05) are indicated with *, ^#^ and ^ for the top, middle and bottom drug, respectively. Data from methylone, α-PVP and MDPV are reproduced from Zwartsen *et al.* 2019.

**Zwartsen, A., Hondebrink, L., Westerink, R.H.S (2019). Changes in neuronal activity in rat primary cortical cultures induced by illicit drugs and new psychoactive substances (NPS) following prolonged exposure and washout to mimic human exposure scenarios. NeuroToxicology 74, 28-39.**
